# EHMTI-0338. The enzymes phosphodiesterase 3 and 5 express activity in the trigeminal ganglion and co-localize with calcitonin gene-related peptide

**DOI:** 10.1186/1129-2377-15-S1-F14

**Published:** 2014-09-18

**Authors:** JC Nordgaard, LS Kruse, M Moeller, C Kruuse

**Affiliations:** 1 University of Copenhagen, Glostrup Hospital Lundbeck Foundation Center for Vascular research PDE group Glostrup Research Park, Glostrup, Denmark; 2 University of Copenhagen, Glostrup Hospital Glostrup Research Park, Glostrup, Denmark; 3 Dept Neuroscience and Pharmacology, Panum Institute Univeristy of Copenhagen, Copenhagen, Denmark; 4 University of Copenhagen, Herlev Hospital Dept Neurology and Lundbeck FOundation Center for Vascular Research PDE group, Herlev, Denmark

## Introduction

The neuropathology leading to migraine pain has centered on an either a vascular or neuronal origin. Sildenafil, a specific inhibitor of phosphodiesterase 5 (PDE5), was the first compound to induce migraine-like headache in a human headache model without a concomitant artery dilatation.

## Aim

In this study we investigated the presence and activity of PDE3 and PDE5 in a key component of the neuronal pathway, the rat trigeminal ganglion. We further addressed a possible cross-talk with a key molecule in migraine pain signaling, calcitonin gene related peptide (CGRP), by investigating cellular co-localization.

## Methods

Analyzes were performed on isolated rat trigeminal ganglion. Localization was done by immunohistochemistry, in situ hybridization, and western blots. Enzymatic assays for cAMP and cGMP hydrolysis were done using scintillation proximity assay.

## Results

We show that PDE3 and PDE5 are present and express activity in the trigeminal ganglia. PDE3 and PDE5 were observed in the majority of neurons in the ganglion. However, only a subset of these PDE3 and PDE5 positive cells contained CGRP.

## Conclusions

Hydrolysis of cyclic AMP was in contrast to cGMP, influenced by both sildenafil and cilostazol, suggesting sildenafil, cilostazol and CGRP may work through a common cAMP pathway resulting in migraine pain.

No conflict of interest.

